# The SIRT2-mediated deacetylation of AKR1C1 is required for suppressing its pro-metastasis function in Non-Small Cell Lung Cancer

**DOI:** 10.7150/thno.39151

**Published:** 2020-01-12

**Authors:** Hong Zhu, Yan Hu, Chenming Zeng, Linlin Chang, Fujin Ge, Weihua Wang, Fangjie Yan, Qinxin Zhao, Ji Cao, Meidan Ying, Yongchuan Gu, Lin Zheng, Qiaojun He, Bo Yang

**Affiliations:** 1 Zhejiang Province Key Laboratory of Anti-Cancer Drug Research, College of Pharmaceutical Sciences, Zhejiang University, Hangzhou, China; 2 Auckland Cancer Society Research Centre, The University of Auckland, Auckland, New Zealand

**Keywords:** acetylation, AKR1C1, SIRT2, non-small cell lung cancer, metastasis

## Abstract

Aldo-keto reductase family 1 member C1 (AKR1C1) promotes malignancy of Non-Small Cell Lung Cancer (NSCLC) by activating Signal Transducer and Activator of Transcription 3 (STAT3) pathway. However, how the pro-metastatic functions of AKR1C1 are switched on/off remains unknown.

**Methods**: Immunoprecipitation and LC-MS/MS analyses were performed to identify the acetylation on AKR1C1 protein, and the functional analyses (*in vitro* and* in vivo*) were performed to depict the contribution of acetylation to the pro-metastatic effects of AKR1C1.

**Results**: Here we report that acetylated AKR1C1 on two lysine residues K185 & K201 is critical to its pro-metastatic role. The acetylation modification has no impact on the canonical enzymatic activity of AKR1C1, while it is required for the interaction between AKR1C1 to STAT3, which triggers the downstream transduction events, ultimately mobilizing cells. Importantly, the deacetylase Sirtuin 2 (SIRT2) is capable of deacetylating AKR1C1, inhibiting the transactivation of STAT3 target genes, thus suppressing the migration of cells.

**Conclusion**: Acetylation on Lysines 185 and 201 of AKR1C1 dictates its pro-metastatic potential both in vitro and in vivo, and the reverting of acetylation by Sirtuin 2 provides potential therapeutic targets for treatment against metastatic NSCLC patients with high AKR1C1 expression.

## Introduction

Metastasis accounts for more than 90% of Non-Small Cell Lung Cancer (NSCLC) related death 1, 2. Although a large number of NSCLC patients have benefited from targeted therapies that constrain the abnormal proliferation of tumor cells, such therapeutic improvement did not equally avail those patients with tumor malignancy, largely due to the lack of intervention targets for the treatment of metastatic NSCLC 3. Current knowledge of NSCLC metastasis primarily focuses on the pro-metastasis roles of cancer stem cells (CSCs) or transcription factors involved in epithelial-mesenchymal transition (EMT) 4-7. However, both CSCs and EMT-related transcription factors remain technically challenging to target 4, 8. Identifying additional druggable targets warrants further studies to fully understand the molecular mechanisms underlying NSCLC metastasis 9.

Among all cancer types, NSCLC harbors the highest expression of aldo-keto reductase family 1 member C1 (AKR1C1) 10. AKR1C1, also known as 20α-HSD, is a member of the human aldo-keto reductase protein family that catalyzes NADP^+^-dependent reduction, and thus plays essential roles in the metabolisms of steroid hormones, prostaglandins and polycyclic aromatic hydrocarbons 11. Previous studies have identified AKR1C1 as a key driver promoting malignancy in various cancers 12-16. Concomitantly, our recent finding uncovered that AKR1C1, by reinforcing the activation of STAT3 pathway, significantly accelerates NSCLC metastasis 10. Furthermore, the experimental depletion of AKR1C1 (by either siRNA or shRNA) could effectively revert the metastasis of cancer cells, as well as their drug resistance 15, 17-19. AKR1C1 has been therefore proposed as a potential target for cancer therapy.

The aldo-keto reductase activities of AKR1C1 provide feasible ways to interfere with its biological functions. A series of enzymatic inhibitors of AKR1C1 have been hence developed, such as 3-bromo-5-phenylsalicylic acid (5-BPSA) 20, 21. Evidence showed that the catalytic activities of AKR1C1 could decrease the susceptibility of cancer cells towards daunorubicin and other chemotherapeutic agents 22. However, whether such enzymatic activities contribute to tumor malignancy remains unclear 23. Our recent study showed that depriving AKR1C1 of its reductase activities (by utilizing 5-BPSA or reductase activity-loss mutants) had little effect on cell motility and STAT3 activation, indicating that the canonical enzymatic activities are dispensable for AKR1C1 to promote NSCLC metastasis 10. We therefore postulate that there might be other regulatory mechanisms underpinning the pro-metastatic effects of AKR1C1.

Post-translational modification (PTM) is known to play fundamental roles in regulating the folding, subcellular localization and functional states of proteins. The PTMs of a protein can also affect its interactions with other cellular molecules, thus profoundly modulating innumerable cellular processes. During our investigation, a high abundance of lysine acetylation (an evolutionarily conserved PTM) was observed in AKR1C1 through liquid chromatography-tandem mass spectrometry (LC-MS/MS). Mounting evidence revealed that non-histone protein acetylation contributes to a myriad of biological functions, ranging from transcriptional regulation to protein-protein interaction^24^-^27^. Counter-acting protein acetylation, the deacetylases such as sirtuins (SIRTs) and zinc-dependent histone deacetylases (HDACs), have been demonstrated to play vital roles in the modulation of cellular processes. We therefore speculated lysine acetylation of AKR1C1 contributes to the metastasis of NSCLC cells, and if valid, this regulatory mechanism entails novel therapeutic intervention.

Our study then identified in AKR1C1 two major acetylated residues, at lysines 185 (K185) and 201 (K201). The lysine acetylation was observed to facilitate the binding of AKR1C1 to STAT3, which activated subsequent transductions to enhance cell mobility. Conversely, deacetylation by sirtuin 2 (SIRT2) abrogated AKR1C1-STAT3 binding, and greatly impaired the metastasis-promoting effects of AKR1C1. In addition, the GEO dataset reveals that the mRNA levels of *SIRT2* were significantly downregulated in the tumor samples of NSCLC patients, compared with those of the normal type. Collectively, these findings not only provide the first evidence that AKR1C1 is acetylated, but also reveal that the acetylation is an important regulatory mechanism underlying the pro-metastatic potential of AKR1C1, and SIRT2-mediated deacetylation may represent a novel therapeutic strategy for NSCLC patients harboring high level of AKR1C1.

## Methods

### Cell Culture

All cell lines were purchased from Cell Bank of the Chinese Academy of Sciences and cultured at 37°C in 5% CO_2_. NCI-H1299, NCI-H460 and PC-9 cells were cultured in RPMI1640 with L-Glutamine and supplemented with 10% FBS (Hyclone). 293FT and Cos7 cells were cultured in DMEM with L-Glutamine and supplemented with 10% FBS. A549 was maintained in F12 medium supplemented with 10% FBS. Both cell lines have been mycoplasma-tested, and authenticated using short tandem repeat (STR) profiling every 6 months.

### Immunofluorescence

Cells were seeded at 24-well plate at a confluence of 50%, allowed to attach overnight, and fixed them with 4% paraformaldehyde for 20 minutes and permeabilized them with 0.1% Triton X-100 (Biofroxx, 1139ML500). After blocking, the primary antibodies were used overnight at 4°C as follows: AKR1C1 (GeneTex, GTX105620), SIRT2 (Sigma-Aldrich, S8447).After washed with PBS three times, cells were incubated for 1 h at room temperature with following appropriate secondary antibodies: Donkey anti-Mouse IgG (H+L) Highly Cross-Adsorbed Secondary Antibody, Alxa Fluor 488 (Invitrogen, 1820538), Donkey anti-Rabbit IgG (H+L) Highly Cross-Adsorbed Secondary Antibody, Alexa Fluor 568 (Invitrogen, 1606268). Nuclei were visualized by staining with DAPI (Sigma-Aldrich, D9542). The immunofluorescence images were captured under a fluorescence microscope (Leica).

### Immunoprecipitation and Western Blot

Whole-cell extracts were lyzed in lysis buffer (25 mM Tris, 150 mM NaCl, 10% Glycerol, 1% NP40, PH=7.4) supplemented with protease inhibitor cocktail (Selleck, S7380). Lysate were boiled for 15 min after additional of SDS sample buffer and separated using SDS-PAGE. For immunoprecipitation, especially for acetylation immunoprecipitation, 4 μM TSA (Selleck, S1045) and 5 mM NAM (Sigma-Aldrich, V900517) were added in the lysis buffer. Immunoprecipitation was carried out either by incubating HA beads (Biotool, B23301) or Flag beads (Biotool, L00425) at 4°C with lysis buffer overnight. Immunoprecipitated protein complexes were washed using wash buffer (25 mM Tris, 150 mM NaCl, 0.2 % NP40, PH=7.4) at least 5 times, boiled in SDS sample buffer for 15 min and detected using Western Blot. The antibodies used as following: AcK (PTM Biolab, PTM101; HuiOu Biotechnology, HOPTM05-02), AKR1C1 (GeneTex, GTX105620 for Western Blot; Santa Cruz, sc-166297, for immunoprecipitation), SIRT2 (Sigma-Aldrich, S8447), p-STAT3(Tyr705) (Cell Signaling Technology, 9145S), STAT3 (Cell Signaling Technology, 9139S), GST (Santa Cruz, sc-138), HA (Diag Biotechnology, db2603), GAPDH (Diag Biotechnology, db1209), β-Actin (Santa Cruz, sc-1615), α-tubulin (Santa Cruz, sc-58666), Flag (Genescript, A00187-100), Sox2 (Santa Cruz, sc-365964), Vimentin (Santa Cruz, sc-80975).

### *In Vitro* Deacetylation Assay

293FT cells were transfected with HA-tagged AKR1C1 (treated with TSA 4 μM and NAM 5 mM for 12 h before harvest) or Flag-tagged SIRT2 for 48 h. Whole-cell extracts were lyzed in lysis buffer, then AKR1C1 or SIRT2 protein was pulled down using the HA/Flag-beads. *In Vitro* deacetylation assay was performed in 50 μL of reaction mixture (PH=8.0) containing 25 mM Tris-HCl, 150 mM NaCl, 5 μg/mL Leupeptin, 20 μg GST-AKR1C1/SIRT2 and HA/Flag-beads for 2 h at 37°C. The reaction mixture was subject to western blot analysis using the anti-acetyllysine antibody.

### RNA extraction and Real-Time qRT-PCR

Total RNA was isolated and purified using the EasyPure RNA Kit according to manufacturer's instructions. 2 μg of RNA was reversely transcribed into cDNA using oligo (dT) priming, followed by SYBR Green real-time PCR. *β-ACTIN* housekeeping gene was used as the endogenous control to normalized the amounts of RNA in each sample. The sequences of oligonucleotide primers were synthesized by Shangya and listed below.

*SOX2*_F:5'-TACAGCATGTCCTACTCGCAG-3'

*SOX2*_R: 5'-CTGCGAGTAGGACATGCTGTA-3'

*TWIST*_F: 5'-GGCATCACTATGGACTTTCTCTATT-3'

*TWIST*_R: 5'-AATAGAGAAAGTCCATAGTGATGCC-3'

*β-ACTIN*_F: 5'-GGTCATCACTATTGGCAACG-3'

*β-ACTIN*_R: 5'-CGTTGCCAATAGTGATGACC-3'

### Gene transfection and RNA interference

Plasmid transient transfection was performed using jetPRIME according to the manufacturer's instructions. The information of the constructs utilized in this manuscript was listed in the Table S1.

For shRNA experiments, 293FT cells were transfected with vector (pLKO.1) or pLKO.1-shAKR1C1 using Lipofectamine 2000. Medium may be changed after 18h, and cell supernatant was collected on Days 3 and 4. The supernatant was filtered through a 0.45-μm filter and stored in -80°C. Target cells were infected by retrovirus supernatant in the presence of 8 μg ml^-1^polybrene every 12h for 3 rounds. Stable cells were selected by using 4 mg ml^-1^puromycin for 3 days. The human short RNA target sequences were listed below.

shAKR1C1-UTR#1: 5'-CCGGGACACAGAGGATGGCTCTATGCTCGAGCATAGAGCCATCCTCTGTGTCTTTTTG-3';

shAKR1C1-UTR#2: 5'-CCGGATGCCATTGGTTAACCAGCAGCTCGAGCTGCTGGTTAACCAATGGCATTTTTTG-3';

shAKR1C1-#1: 5'-CCGGAAGCTTTAGAGGCCACCAAATCTCGAGATTTGGTGGCCTCTAAAGCTTTTTTTG-3';

shAKR1C1-#2: 5'CCGGGCCACCAAATTGGCAATTGAACTCGAGTTCAATTGCCAATTTGTGGCTTTTTG-3';

shSIRT2-#1:5'-CCGGCCTGCTCATCAACAAGGAGAACTCGAGTTCTCCTTGTTGATGAGCAGGTTTTTG-3';

shSIRT2-#2: 5'-CCGGGCTAAGCTGGATGAAAGAGAACTCGAGTTCTCTTTCATCCAGCTTAGCTTTTTG-3'.

### Transwell assay

The migration ability of NSCLC cells was assessed by transwell assay. In brief, cells(2×10^5^) were plated on the upper chamber (Corning, 353504) of the transwell inserts in serum-free medium, which was situated in a well of a 24-well culture plate and immersed in the 600 μL medium supplemented with 10% FBS. After incubation for 24 h, non-migrating cells were on the upper chamber side of the filters while the migrated cells were on the lower side of the membrane. Cells were photographed and counted with an inverted microscope after 0.1% crystal violet staining (Yuanhang Chem, YHSJ-01-92).

### In-gel digestion for mass spectrometry analysis

**Figure 1:**
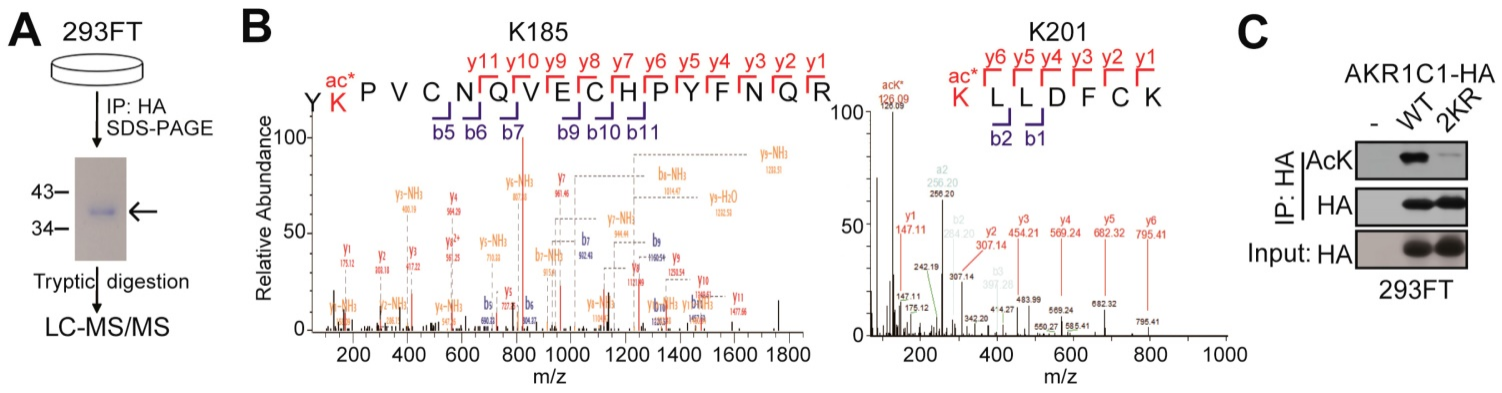
** AKR1C1 is acetylated at Lysines 185 and 201. (A)** Schematic representation of mass spectrometry process. HA-tagged AKR1C1 was transfected into 293FT cells, and AKR1C1 was purified by immunoprecipitation with anti-HA beads. The immunoprecipitated AKR1C1-HA was subjected to SDS-PAGE, and the band corresponding to AKR1C1 was digested in-gel with trypsin. The labeled peptides were analyzed by LC-MS/MS. **(B)** Identification of AKR1C1 K185 and K201 acetylation using mass spectrometry analysis. **(C)**Transfection of 2KR mutants significantly decreased AKR1C1 acetylation. Acetylation of ectopically expressed AKR1C1-WT/2KR in 293FT cells was analyzed.

To identify the acetylated sites of AKR1C1, HA tagged-AKR1C1 plasmid was transfected into 293FT cells and treated with 4 μM TSA and 5 mM nicotinamide for 12 h before harvest. Cells were collected and lyzed in 1% NP40 lysis buffer with 4 μM TSA and 5 mM nicotinamide, and the cell lysate was immunoprecipitated with the anti-HA beads at 4°C overnight. The immunoprecipitated AKR1C1-HA was separated using SDS-PAGE. And the bands corresponding to AKR1C1 were excised from the gel and subjected to trypsin digestion and mass spectrometry. Modification sites were performed according to Figure 1 by PTM BioLab (Hangzhou, China).

### Enzyme activity assay

Michaelis-Menten constants for both enzymes were determined in 0.1 M potassium phosphate (pH=6.7), 0.1 mM NADP^+^ (Sangon Biotech, A600760-0250), different concentrations of substrate, S-tetralol (Santa Cruz, sc-253491) and 20 μg AKR1C1-WT/2KR protein. The assay of catalytic activity was spectrophotometrically carried out by measuring the rate of change in NADPH absorbance (ε_λ340_ = 6220 M^-1^.cm^-1^) over time. The inhibitor 3-bromo-5-phenylsalicylic acid, 5-BPSA (Cayman, 13574) was dissolved in dimethyl sulfoxide (DMSO) and used in the assay as a system control.

K_m_ and V_max_ vaules were determined from the plots of the initial velocities versus the concentration of substrate, using GraphPad Prism Version 6.0 for windows.

### Enzyme expression and purification

The pGEX-4T1-AKR1C1-WT/2KR construct were transferred into the E.coli BL21 (DE3). And the expression of protein was induced by IPTG (Mai bio, 120820) at a final concentration of 0.3 mM at 25°C for 16 h. The induced proteins were purified by the affinity chromatography (Glutathione S-transferase (GST)-fusion protein affinity binding to Glutathione-Sepharose (Sangon Biotech, c600031-0010)) followed by thrombin cleavage as described in the GST Gene Fusion System Handbook. The concentrated proteins were quantified by Bio-Rad Bradford Protein assay kit with wavelength of OD_595_ nm. The purified protein was checked by SDS-PAGE followed by Coomassie Blue Staining (Ourchem, 6104-58-1). The purified protein was stored at -80°C after addition of 50% glycerol.

### *In vivo* metastatic foci analyses

BALB/c-Nude mice (4-5 weeks of age, female) were injected with 400×10^4^ cells in 200 μL medium via tail vein. After 60 days, mice were sacrificed and their livers and lungs were dissected, fixed with phosphate-buffered neutral formalin and prepared for standard histological examination. The animal studies were approved by the Animal Research Committee at Zhejiang University, with ethical approval number IACUC-18121, and all experimental protocols were conducted in accordance with institutional guidelines.

### Statistical analysis

Experiments were performed in triplicates and repeated at least three times otherwise as indicated. Data are presented as mean ± SD from 3 independent experiments. Comparisons between two groups were performed using two-tailed Student's t-test. Differences between multiple groups were determined using One-way ANOVA. *p* < 0.05 was considered significant (*: *p* < 0.05; **: *p* < 0.01; ***: *p* <0.001).

## Results

### AKR1C1 is acetylated at lysines 185 and 201

In order to study the PTMs on AKR1C1 proteins, we first performed immunoprecipitation (IP) on AKR1C1, which was then subject to proteolytic digestion and LC-MS/MS analysis (Figure 1A). The results not only revealed that AKR1C1 was an acetylated protein, but also located its two prominent acetylation sites at lysines K185 and K201 (Figure 1B). To further verify the acetylation modification, we performed IP assays on AKR1C1 using an anti-acetyllysine antibody. As shown in Figure S1A, ectopically expressed AKR1C1 proteins in 293FT and Cos7 cells were both acetylated. More importantly, the acetylation of endogenous AKR1C1 in NSCLC PC-9 cells was also observed (Figure S1B). After co-treatment of both cells with SIRT inhibitor nicotinamide (NAM) and suberoylanilidehydroxamic acid (SAHA), an inhibitor of zinc-dependent HDACs, the level of acetylated AKR1C1 significantly increased (Figure S1C). To directly confirm the sites of acetylation, acetylation-deficient mimics were constructed by mutating both lysines K185 and K201 to arginine (2KR). The transfection of 2KR in 293FT cells significantly reduced the acetylation level of AKR1C1 (Figure 1C).

Taken together, these findings corroborate that AKR1C1 is an acetylated protein with two acetylated residues at lysines K185 and K201.

### Acetylation is fundamental for the pro-metastatic ability of AKR1C1 in NSCLC

**Figure 2:**
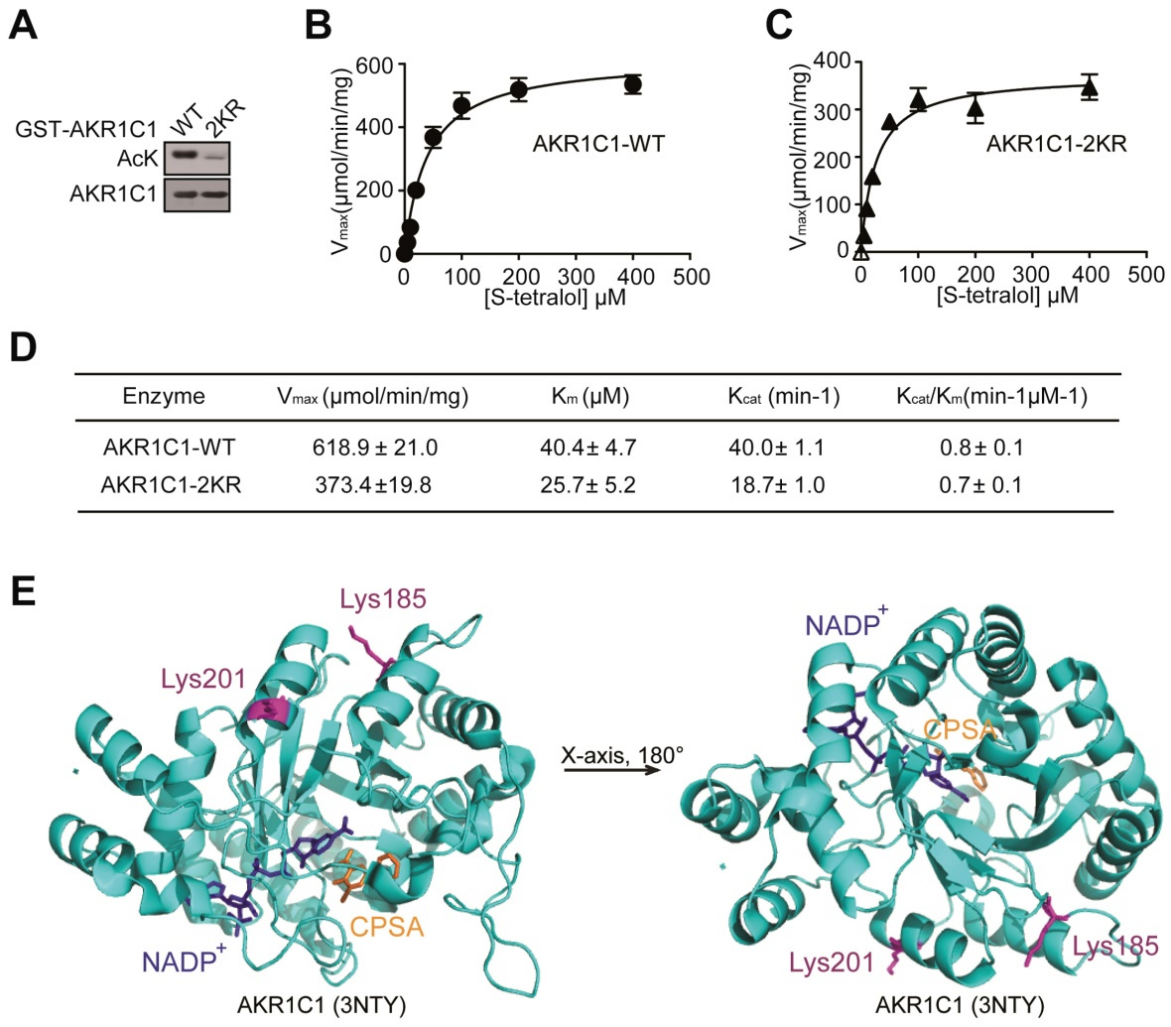
** Acetylation has no effects on the reductase activity of AKR1C1. (A)** Bacterially expressed GST-AKR1C1-2KR protein had a dramatically decreased acetylation level compared with GST-AKR1C1-WT. **(B-C)**Michaelis-Menten Plots for S-tetralol catalyzed by AKR1C1-WT and AKR1C1-2KR, each point represents the mean ± SD of at least three experiments. **(D)**Kinetic parameters for S-tetralol catalyzed by AKR1C1-WT and AKR1C1-2KR were acquired from 100 mM sodium phosphate buffer, at PH7.0, with 200 μM NADP^+^. **(E)** The position of lysine185/201 in the AKR1C1-NADP^+^-3-chloro-5-phenylsalicylic acid (CPSA) complex (RCSB PDB: 3NTY). The coenzyme NADP^+^ and inhibitor CPSA that interact with AKR1C1 are depicted using stick models.

Mounting evidence has revealed that acetylation and deacetylation of a given protein is critical for regulating its biological functions, ranging from protein stability to catalytic activities 26-28, therefore we investigated whether the acetylation of AKR1C1 would influence its enzymatic activity. The wild-type (WT) and 2KR mutant (Figure 2A) were purified and the catalytic activities between the two proteins were compared in the presence of S-tetralol as a substrate for oxidase activity. As shown in Figure 2B-D, the mutant proteins exhibited similar catalytic activities, as indicated by the close or comparable values of K_cat_/K_m_, V_max_, K_cat_and K_m_. This finding was further supported by the crystal structure of AKR1C1 (RCSB PDB, 3NTY, https://www.rcsb.org/structure/3NTY), wherein the lysine-acetylation residues (Lys185 and Lys201) were associated with neither enzymatic co-factor NADP^+^ nor AKR1C1 catalytic inhibitor 5-BPSA (Figure 2E). To assess whether the acetylation affect AKR1C1 protein stability, we compared the protein half-life between AKR1C1 WT and 2KR mutant, and little difference was found (Figure S2). These observations collectively suggest that the acetylation of AKR1C1 influences neither its enzymatic function nor its protein turnover.

**Figure 3:**
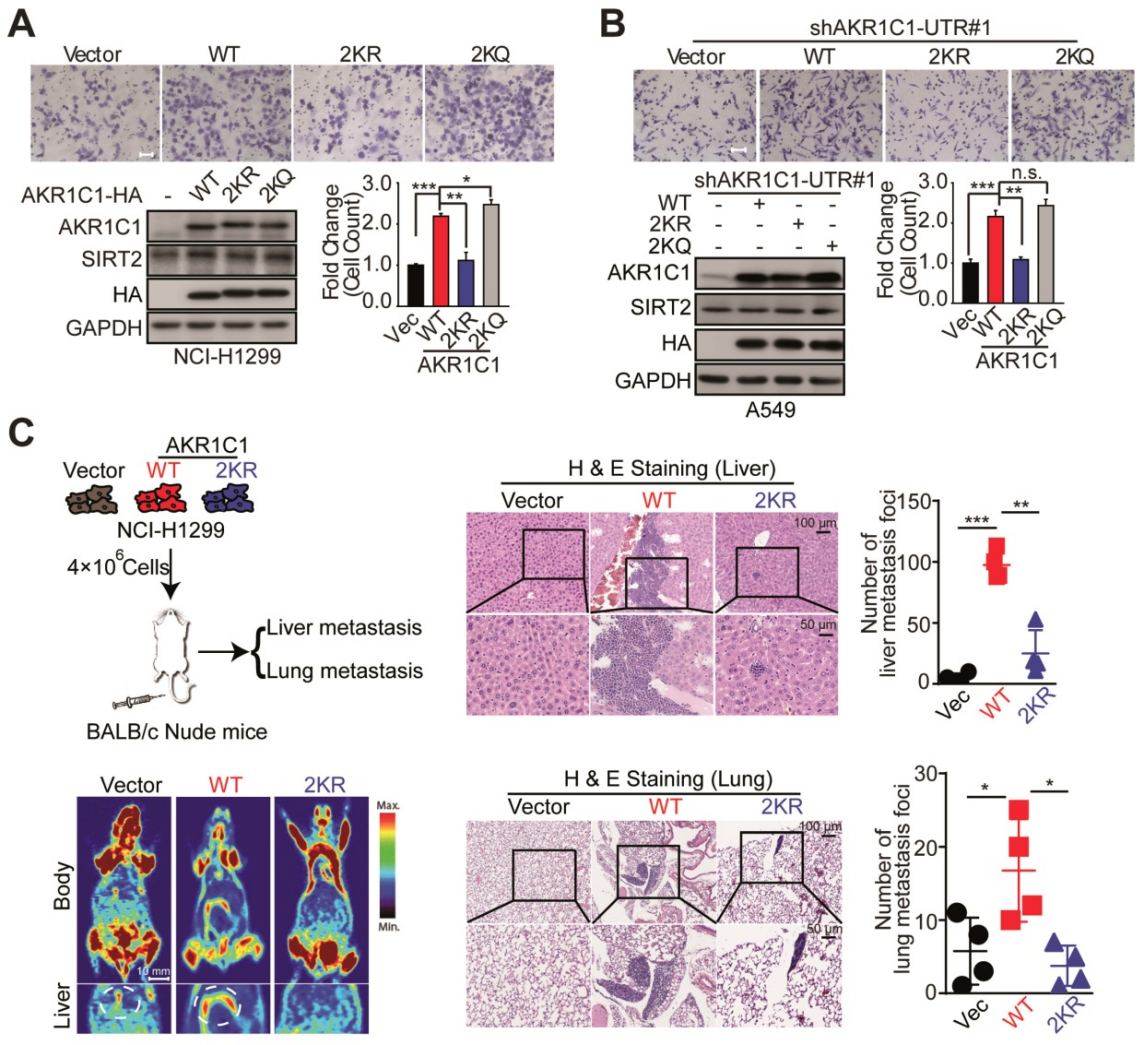
** Acetylation of AKR1C1 is fundamental for its pro-metastastic ability in NSCLC. (A)** A transwell assay was used to analyze the migration activity of NCI-H1299 cells transfected with wild-type and double-mutant-type (2KR and 2KQ) AKR1C1 (n=3; *: *p*<0.05, **: *p*<0.01, ***: *p*<0.001). Scale Bar, 200 μm; Western Blot analysis showing the transfected efficiency of AKR1C1s. **(B)** The effects on cell migration of wild-type and double-mutant-type (2KR and 2KQ) AKR1C1 were examined in A549 cells depleted of endogenous AKR1C1 (n=3; **: *p*<0.01, ***: *p*<0.001). Scale Bar, 100 μm; Western Blot analysis showing the transfected efficiency of AKR1C1s.** (C)** Over-expressed AKR1C1 in NCI-H1299 cells promoted liver and lung metastasis in nude mice models. Representative micrographs with metastatic nodules were shown by micro-pet and hematoxylin and eosin staining, and the number of metastatic nodules was counted under a microscope. Scale Bar, 10 mm, 100 μm and 50 μm; Statistical significance was determined by Student's t-test (n=4; *:* p*<0.05,**: *p*<0.01, ***: *p*<0.001).

Given the fact that AKR1C1 was a pro-metastatic factor, we were then prompted to investigate whether the acetylation contributes to such metastasis-promoting ability. As shown in Figure 3A, in NCI-H1299 cells harboring low level of AKR1C1, the exogenous transfection of AKR1C1-WT greatly induced the migration of NCI-H1299 cells, in line with our recent finding 10, whereas 2KR non-mimics significantly blunted cell mobility. Moreover, the acetyl-lysine mimics (2KQ) demonstrated even stronger ability to promote cell migration. Subsequently, we attempted to further confirm these results in A549 cells in which the expression level of AKR1C1 was elevated (Figure S3 and S4A). The endogenous AKR1C1 was knocked down to minimize its impact through shRNA targeting the untranslated regions before WT, 2KR and 2KQ mutants were introduced into the cells, respectively (Figure S3A). Consistent with the above results, AKR1C1-2KR lost its pro-metastatic effect, while 2KQ mutant withholding strong metastasis-promoting function (Figure 3B). To replicate the findings that acetylation of AKR1C1 is fundamental for the metastasis of NSCLC *in vivo*, H&E staining was used to evaluate tumor metastasis in nude mice intravenously injected with NCI-H1299 cells stably expressing Vector, AKR1C1-WT and AKR1C1-2KR (Figure S3B). Mice injected with NCI-H1299 cells expressing AKR1C1-WT, but not those injected with cells expressing AKR1C1-2KR, showed significantly increased metastatic foci in the liver and lung after injection (Figure 3C).

Taken together, these data validate that the acetylation modification conferred AKR1C1 with its pro-metastatic capabilityboth *in vitro* and *in vivo*.

### SIRT2 interacts with and deacetylates AKR1C1

**Figure 4:**
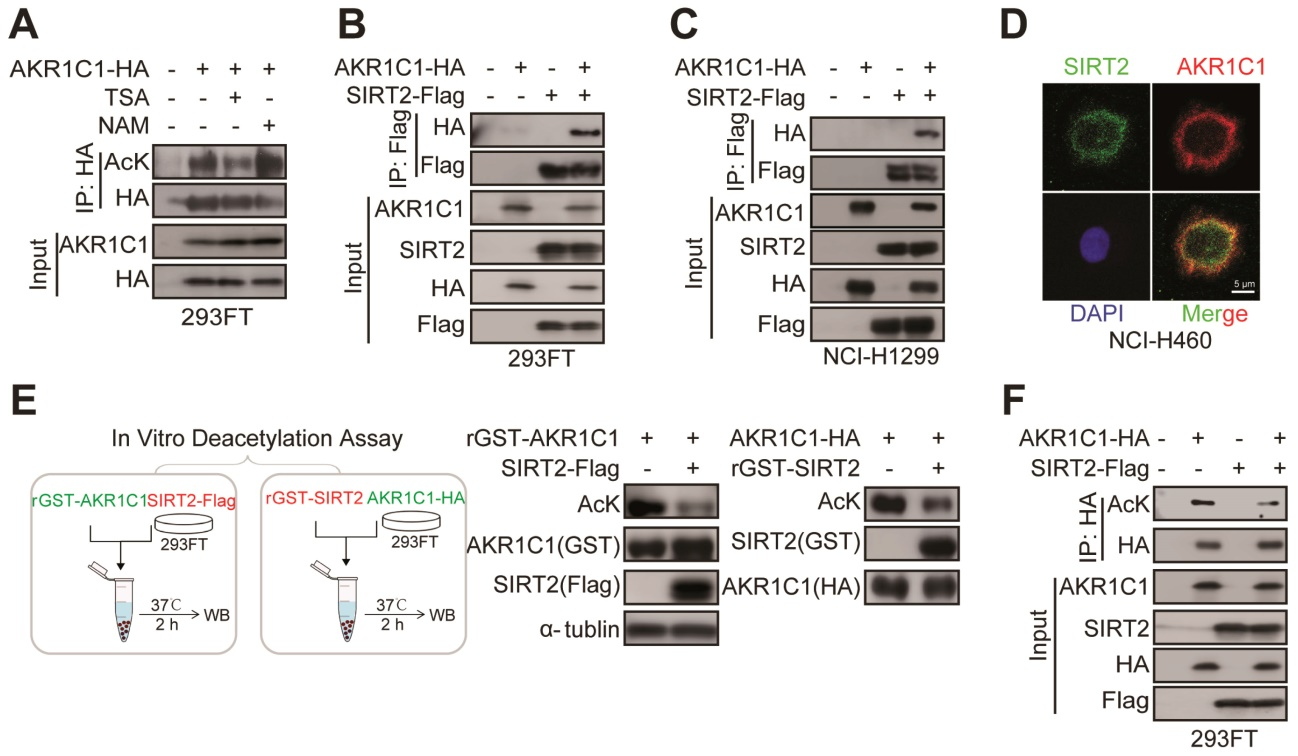
** SIRT2 interacts with and deacetylates AKR1C1. (A)** NAM, but not TSA, increased AKR1C1 acetylation. HA-tagged AKR1C1 was transfected into 293FT cells with either NAM or TSA treatment, followed by IP Western Blot analyses. **(B-C)** Association of AKR1C1 with SIRT2. HA-tagged AKR1C1 and Flag-tagged SIRT2 were transfected into 293FT cells and NCI-H1299 cells either singly or in combination. The interaction between AKR1C1 and SIRT2 was detected by IP Western Blot analyses. **(D)** Co-localization of endogenous AKR1C1 and SIRT2 demonstrated by immunofluorescence of SIRT2 (Green) and AKR1C1 (Red) in NCI-H460 cells. DAPI (Blue) was used to visualize nuclei. Scale Bar, 200 μm and 50 μm.** (E)** SIRT2 deacetylated AKR1C1 *in Vitro*. In Vitro deacetylation assay of recombinant GST-AKR1C1 mixed with purified protein Flag-tagged SIRT2, and GST-SIRT2 with HA-tagged AKR1C1 at 37°C for 2 h. The reaction mixtures were subjected to SDS-PAGE followed by immunoprecipitation using an anti-acetyllysine antibody. **(F)** AKR1C1 is deacetylated by SIRT2. HA-tagged AKR1C1 was expressed in 293FT cells together with Flag-tagged SIRT2. AKR1C1 proteins were purified by HA beads, and acetylation level was detected by Western Blot analyses.

Following our finding that AKR1C1 is an acetylated protein and acetylation is fundamental for the pro-metastasis ability of NSCLC, we sought to identify its effective deacetylase(s). 293FT or PC-9 cells were treated with either NAM, a general inhibitor of SIRTs, trichostatin A (TSA), an inhibitor of zinc-dependent HDACs, or Tubacin, a specific inhibitor of HDAC6 29. Intriguingly, we found that only NAM treatment greatly increased the acetylation level of AKR1C1, whereas TSA or Tubacin mediation showed no noticeable effect (Figure 4A, Figure S1B and Figure S4B), indicating that SIRTs family was preferentially involved in the deacetylation of AKR1C1. Given that AKR1C1 is located in cytoplasm, we asked whether SIRT2, the only predominately cytoplasmic-located member of SIRTs, could interact with and deacetylate AKR1C1. To assess the interaction between SIRT2 and AKR1C1, we performed co-immunoprecipitation (co-IP) from 293FT cells transfected with ectopically expressed HA-tagged AKR1C1 and Flag-tagged SIRT2, and observed that AKR1C1 could be indeed co-precipitated from cell lysates together with SIRT2 by anti-Flag antibody (Figure 4B). In addition, the purified glutathione S-transferase (GST)-tagged AKR1C1 fusion protein (GST-AKR1C1) was found to interact with SIRT2 but not HDAC6 from NSCLC PC-9 cell lysates (Figure S4C). This finding not only supported the observed interaction between SIRT2 and AKR1C1, but also indicated that HDAC6 was not involved in the regulation of AKR1C1, which was in line with the aforementioned results utilized HDAC6 inhibitor, Tubacin (Figure S1B and S4B). Similar protein-protein interaction between AKR1C1 and SIRT2 was also observed from another NSCLC NCI-H1299 cells (Figure 4C), suggesting the formation of AKR1C1-SIRT2 complex in lung cancer models. Consistently, immunofluorescence (IF) staining of endogenous AKR1C1 and SIRT2 revealed their co-localization (preferentially in cytoplasm) in NSCLC NCI-H460 cells (Figure 4D) harboring high levels of SIRT2 and AKR1C1 (Figure S4A). Taken together, these findings show that SIRT2 could physically engage AKR1C1.

To further interrogate whether AKR1C1 is the substrate for the enzymatic activity of protein deacetylase SIRT2, two types of *in vitro* deacetylation assays were employed to determine: 1) the deacetylating effects of Flag-tagged SIRT2, immunoprecipitated from cell lysate, on the purified GST-AKR1C1 protein; 2) the deacetylating effects of purified recombinant GST-SIRT2 on HA-tagged AKR1C1, immunoprecipitated from cell lysate. As displayed by Figure 4E, both purified GST-AKR1C1 and precipitated HA-AKR1C1 were remarkably deacetylated by SIRT2. Concordantly, overexpression of SIRT2 in 293FT cells resulted in major reduction of acetylation levels in exogenous AKR1C1 proteins (Figure 4F). Notably, SIRT1 was found to interact with but failed to deacetylate AKR1C1 (Figure S4D-E).

Collectively, these results represent SIRT2 as the protein deacetylase for AKR1C1 through physical interaction.

### SIRT2 suppresses the metastatic-promoting effects of AKR1C1 in NSCLC by deacetylation

**Figure 5:**
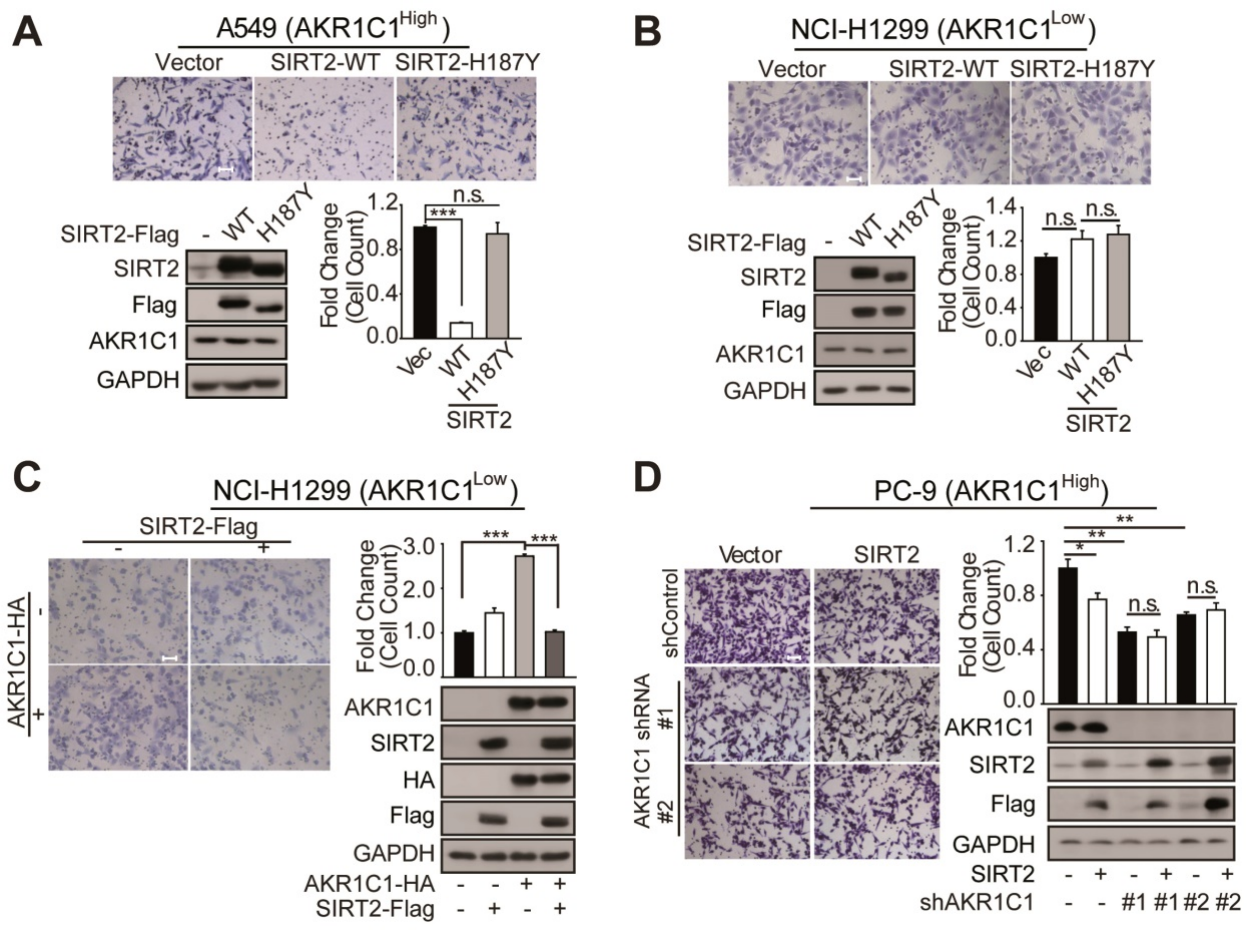
** SIRT2 suppress the metastatic ability of AKR1C1 in NSCLC. (A-B)** Migration assays in A549 and NCI-H1299 cells after transfecting with vector, SIRT2-WT and SIRT2-H187Y plasmids (n=3; ***: *p*<0.001). Left Scale Bar, 200 μm; right Scale Bar, 100 μm; Western Blotting analysis showing the transfected efficiency of SIRT2. **(C)** A transwell assay was used to analyze the role of SIRT2 in reversing the pro-metastasis ability of AKR1C1 (n=3; ***: *p*<0.001). Scale Bar, 200 μm; Western Blotting analysis showing the transfected efficiency of AKR1C1 and SIRT2. **(D)**Transwell assay showing migration abilities of AKR1C1 knockdown PC-9 cells transfected with vector or SIRT2 (n=3; *: *p*<0.05, **: *p*<0.01). Scale Bar, 200 μm; Western Blot analysis showing the knockdown efficiency of AKR1C1 and transfected efficiency of SIRT2.

Given our finding that SIRT2 interacted with and deacetylated AKR1C1, we next assessed the negative influence of SIRT2 on AKR1C1-induced metastasis. Exogenous transfection of SIRT2 into A549 cells markedly constrained cell migration, while H187Y deacetylase-defective SIRT2 failed to suppress cell motility (Figure 5A). In addition, SIRT2 knockdown was observed to enhance the migration of A549 cells (Figure S5). Together, these results suggest that SIRT2 inhibits cell migration in a deacetylase activity-dependent manner.

We subsequently sought to interrogate how the protein level of AKR1C1 could affect the regulatory role of SIRT2 on cell motility. We found that SIRT2 exerted little effect on NCI-H1299 cells possessing low protein level of AKR1C1 (Figure 5B). However, the exogenous transfection of AKR1C1 into NCI-H1299 cells markedly restored the suppressive effects of SIRT2 on metastasis, as evidenced by the great loss of migrated cells in SIRT2- and AKR1C1-cotransfection samples, compared with that in AKR1C1-overexpressed group (Figure 5C). Conversely, this metastasis-suppression effect of SIRT2 was significantly dampened when we knocked down AKR1C1 using two different shRNA sequences in PC-9 cells (Figure 5D), further denoting the functional link between SIRT2 and AKR1C1 in NSCLC cell motility regulation.

Collectively, these findings show that SIRT2 could downregulate AKR1C1-induced cell migration in NSCLC, by reverting its acetyl-lysine modification.

### K185/K201 acetylation plays a signaling role in activating STAT3 pathway in NSCLC cells

**Figure 6:**
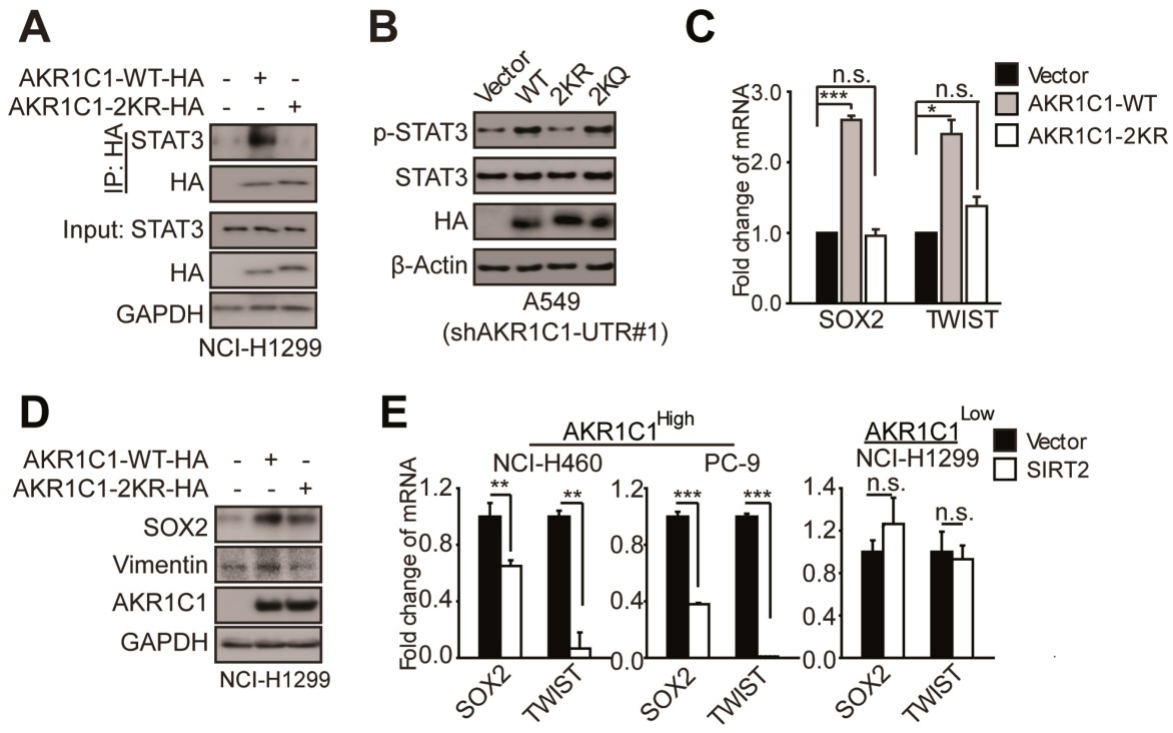
** Acetylation of AKR1C1 plays a signaling role in activating STAT3 pathway in NSCLC. (A)**AKR1C1-WT interacts with STAT3 while AKR1C1-2KR abolishes it.**(B)**Western Blot analysis of p-STAT3 and STAT3 in shAKR1C1-UTR-A549 cells transfected with wild-type and mutant-type (2KR and 2KQ) AKR1C1 for 2 days. **(C)** Quantitative real-time PCR for analyzing the mRNA levels of *SOX2* and *TWIST* in NCI-H1299 cells stably transfected with an empty vector/AKR1C1-WT/AKR1C1-2KR for 48 h (n=3; *: *p*<0.05, ***: *p*<0.001).**(D)** Western Blot analysis showing the SOX2 and Vimentin protein abundance in different groups in viral infected NCI-H1299 cells. **(E)**Quantitative real-time PCR for analyzing the mRNA levels of *SOX2* and *TWIST* in NCI-H460/PC-9/NCI-H1299 cells transfected with SIRT2 or empty vector for 48 h (n=3; **: *p*<0.01, ***: *p*<0.001).

Our previous study has demonstrated that AKR1C1 induces NSCLC metastasis by activating STAT3 pathway, as indicated by the increased phosphorylation levels and transcriptional activities of STAT3 10. Similar STAT3-activation effect by AKR1C1 was also observed by a recent study conducted by Chang *et al* 30. Moreover, several lines of evidence showed that STAT3 signaling indeed plays essential roles in the malignant progression of NSCLC and also the other types of tumor 31-32. In the light of such insight, we sought to investigate whether K185/K201 acetylation of AKR1C1 contributes to STAT3 signaling, and ultimately promotes NSCLC cell metastasis. First, we employed co-IP to examine the binding affinity of AKR1C1-WT and 2KR to STAT3. The results showed that AKR1C1-WT could soundly engage STAT3, whereas little interaction was detected between 2KR mutant and STAT3 (Figure 6A). We then examined the phosphorylation levels of STAT3 in AKR1C1-WT or 2KR-transfected A549 cells depleted of endogenous AKR1C1 (Figure S6A). As shown in Figure 6B, consistent with our previous findings 10, AKR1C1-WT introduction could enhance the p-STAT3 levels. By contrast, 2KR mutant failed to increase the phosphorylation of STAT3. Moreover, acetyl-lysine mimic 2KQ mutant sustained the p-STAT3-induction ability (Figure 6B; Figure S6B), suggesting that the acetylation of K185 and K201 residues in AKR1C1 indeed regulates STAT3 pathway.

To corroborate these findings, we examined the transactivation of STAT3 target genes, and found that the mRNA and protein levels of those metastasis-related target genes (e.g. *SOX2* and *TWIST*) were significantly increased in the presence of AKR1C1-WT, whereas 2KR mutant produced little effect (Figure 6C-D). These data attest the key role of AKR1C1 acetylation in STAT3 activation.

Since we have demonstrated earlier that SIRT2 is essential for the regulation of AKR1C1 acetylation and consequential cellular migration, we postulated that SIRT2 might also modulate the expression of STAT3 target genes by deacetylating AKR1C1. The results showed that SIRT2 overexpression in NCI-H460 and PC-9 cells with high AKR1C1 volume significantly dampened the transactivation of STAT3 target genes (Figure 6E), according with our previous data from cell motility assays (Figure 5). On the contrary, in NCI-H1299 cells with low expression levels of AKR1C1, the ectopic introduction of SIRT2 imposed little effect on STAT3 target genes (Figure 6E).

**Figure 7:**
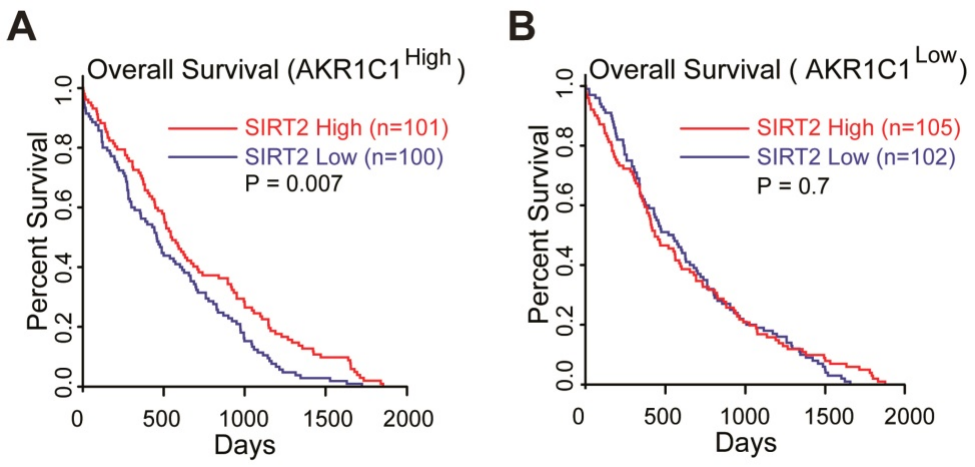
***SIRT2* expression levels correlated with longer overall survival (OS) in AKR1C1^high^ NSCLC patients (data from TCGA database: all of data in the lung adenocarcinoma and lung squamous cell carcinoma). (A)** The OS curves of *SIRT2*^low^ (n = 100) and *SIRT2*^high^ (n = 101) NSCLC patients harboring high expression of *AKR1C1* (*AKR1C1*^high^). **(B)** The OS curve of *SIRT2*^low^ (n = 102) and *SIRT2*^high^ (n = 105) in AKR1C1 low-expressing NSCLC patients (*AKR1C1*^low^).

Collectively, these data suggested that the acetylation on K185 and K201 residues of AKR1C1 plays critical roles in activating STAT3, while SIRT2 reverts this signaling pathway in an AKR1C1-dependent manner. This finding provides the mechanistic framework by which AKR1C1 acetylation promotes the metastasis of NSCLC cells, and represents SIRT2 as a down-regulator of NSCLC metastasis through the deacetylation of AKR1C1. This finding also concurs with its clinical relevance: 1) the normal lung tissues harbor significantly higher level of SIRT2 than NSCLC type (Figure S7A, data from GEO database GSE40275); 2) those high risk NSCLC patients possess much lower mRNA level of SIRT2 (Figure S7B). More importantly, TCGA database was utilized to analyze the relationship between *SIRT2* expression level and Overall Survival (OS) in *AKR1C1*-highly-expressed (*AKR1C1*^high^) and *AKR1C1*-lowly-expressed (*AKR1C1*^low^) NSCLC patients, respectively. The statistical analysis of survival was performed by R package survival and survminer. In line with the aforementioned data, the influence of *SIRT2* on the Overall Survival (OS) may only exist in *AKR1C1*-highly-expressed (*AKR1C1*^high^) NSCLC patients, as indicated by the prolonged OS in *SIRT2*^high^ sub-population (Figure 7A). In the contrast, for those patients harboring lower levels of *AKR1C1* (*AKR1C1*^low^), there was no significant difference between the *SIRT2*^high^ and *SIRT2*^low^ sub-populations (Figure 7B).

## Discussion

Despite the recent development of intervention treatment, NSCLC remains the leading cause of cancer-associated death worldwide, and the high mortality is largely due to the development of metastasis 1-3. Our previous study showed that AKR1C1 promotes metastasis and predicts poor prognosis in patients with NSCLC, but the underlying mechanism(s) regulating the pro-metastatic effect of AKR1C1 remains incompletely understood 10. In this study, we have demonstrated for the first time that AKR1C1 is acetylated at sites of lysines 185 and 201. By employing both acetylation-deficient and acetylation-mimicking mutants of AKR1C1 (2KR and 2KQ), we corroborated that the acetylation state of AKR1C1 determines its metastasis-promoting functions in NSCLC cells both* in vitro* and* in vivo*. In addition, we elucidated that SIRT2 could directly bind to and deacetylate AKR1C1, resulting in the suppression of cell motility in NSCLC with high level of AKR1C1.

One additional finding of present study is that the acetylation of AKR1C1, while modulating the pro-metastatic function of AKR1C1, imposes little effect on the catalytic activity of AKR1C1. This result is consistent with our prior findings: 1) the canonical enzymatic activity is dispensable for AKR1C1 in promoting NSCLC metastasis; 2) the two paralogues of AKR1C1, namely, AKR1C2 and AKR1C3, although possessing similar catalytic activities and biological functions 15, 16, 33, yet minimally contribute to the malignancy of NSCLC 10. However, Matsumoto *et al.* reported that the inhibition of enzymatic activity of AKR1C1 could suppress the invasion potential of bladder cancer cells 17. The seemingly contradicting conclusions of these two independent studies may arise from the different cellular context of the two types of cancer models, as well as the varied choices of AKR1C1 inhibitors. Matsumoto *et al* utilized flufenamic acid (FFA) with IC_50_ value of 6.0 µM 28, whereas we chose a much more potent inhibitor of AKR1C1, 5-BPSA, with IC_50_ value of 0.5 µM 21. In addition, being a nonsteroidal anti-inflammatory drug, FFA could induce other cellular targets that possibly contributed to the anti-metastatic effects. Therefore, we conclude that the enzymatic activity of AKR1C1 does not interfere with the metastasis of NSCLC cells. In addition, the metastasis-driving acetylation of AKR1C1 is not necessary for its enzymatic activity, further highlight the advantage of the acetylation-modulation as a promising intervention strategy for NSCLC metastasis, since the deprivation of acetylation of AKR1C1 would impose no effect on its catalytic activity which is necessary for the physiology function.

NAD^+^-dependent deacetylase SIRT2 is extensively involved in tumor progression, playing drastically different roles in various tumor models, dictated by the substrate(s) of SIRT2 deacetylase activity 27, 34-36. The oncogenic role of SIRT2 has been identified in basal-like breast cancer cells and hepatocellular carcinoma 27, 37, and the interruption of SIRT2 catalytic activity was reported to potentially target a subset of c-Myc-driven cancers 38. In contrast, SIRT2 was also observed to be tumor-suppressive: Kim *et al* found that aged SIRT2 knockout mice have increased tumor incidence compared with WT controls 39; Fiskus *et al.* demonstrated that SIRT2 inhibits the peroxidase activity of peroxiredoxin, and thus sensitizes breast cancer cells to intracellular DNA damage and cell death 40. Our present study extends the understanding of the role of SIRT2 in cancer by identifying its downregulating effect on the metastasis potential of NSCLC cells with high expression level of AKR1C1. In line with our finding, TCGA and GEO database (GSE40275) mining showed that the expression of SIRT2 was much lower in tumor samples of high-risk NSCLC patients.

Furthermore, AKR1C1 is aberrantly highly expressed in NSCLC cells, with > 20 fold change in either mRNA or protein levels in comparison to the normal types, probably caused by the influence of PM2.5 on a large population 10, 41, 42. And among the AKRCs family, only AKR1C1 is high correlated with prognosis of NSCLC patients. Interestingly, SIRT2-mediated deacetylation is highly specific for AKR1C1. As we found that although the other two paralogues AKR1C2 and C3 share highly similarity on the sequence and structure with AKR1C1, and even also have the acetylation modification, yet SIRT2 failed to revert the modification on AKR1C2 and C3 (Figure S8). Therefore, by specifically regulating the acetylation level rather than the protein expression level (Figure S2 and S9) of AKR1C1, SIRT2 activation could be a beneficial strategy to inhibit or prevent the AKR1C1-caused metastasis of NSCLC.

**Figure 8:**
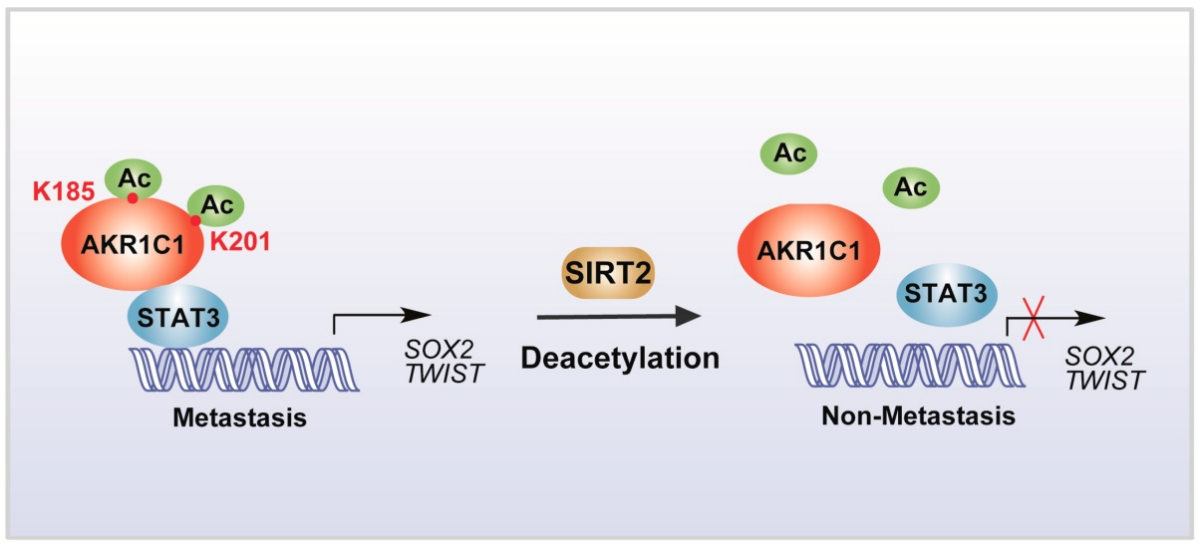
Scheme for the mechanism of acetylation of AKR1C1 and its role in the metastasis of NSCLC.

In summary, our study identified that the acetylation on lysine residues K185 and K201 determines the pro-metastatic effects of AKR1C1. Deacetylase SIRT2 could bind to AKR1C1 and revert the acetylation modification, and thus interrupts STAT3 signaling followed by its downstream transductions, ultimately suppressing the pro-metastatic function of AKR1C1 in NSCLC models (Figure 8). Such mechanistic findings not only broaden the understanding of AKR1C1 as an acetylated protein, but also highlight this acetylation as a major regulator of the pro-metastasis potential of AKR1C1. Inhibiting acetylation-facilitated STAT3 signaling could reduce the metastasis from NSCLC cells, and hence provide a potentially promising approach for the treatment of NSCLC malignancy in AKR1C1-postive patients.

## Conclusions

In summary, acetylation on lysines 185 and 201 of AKR1C1 dictates its pro-metastatic potential, and the reverting of acetylation by Sirtuin 2 provides potential therapeutic targets for treatment against metastatic NSCLC patients with high AKR1C1 expression.

## Supplementary Material

Supplementary figures and table.

## Abbreviations

AKR1C1: Aldo-keto reductase family 1 member C1
SIRT2: Sirtuin 2
NSCLC: Non-Small Cell Lung Cancer
STAT3: Signal Transducer and Activator of Transcription 3
PTM: Post-translational modification
NAM: nicotinamide
HDAC: Histone Deacetylases
TSA: Trichostatin A
GST: glutathione S-transferase
TCGA: The Cancer Genome Atlas
5-BPSA: 3-bromo-5-phenylsalicylic acid
CPSA: 5-Phenyl,3-chlorosalicylic acid
UTR: Untranslated Region
CHX: Cycloheximide
