# Supplementary material for: The SIRT2-mediated deacetylation of AKR1C1 is required for suppressing its pro-metastasis function in Non-Small Cell Lung Cancer
Source: Theranostics. 2020 Jan 12;10(5):2188–200. doi: 10.7150/thno.39151 (PMC7019158; doi:10.7150/thno.39151)
Supplement: Supplementary file 1 — Supplementary figures and table. [file thnov10p2188s1.pdf]

Supplementary Figures and Legends:

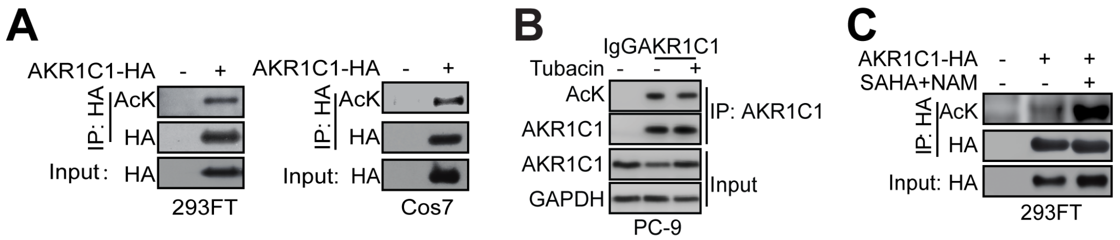

Figure S1. AKR1C1 is an acetylated protein. (A) Exogenous AKR1C1 is acetylated in 293FT cells and Cos7 cells. Acetylation of immunoprecipitated HA-tagged AKR1C1 from 293FT cells and Cos7 cells. **(B)** Acetylation of immunoprecipitated endogenous AKR1C1 from NSCLC PC-9 cells, with or without the exposure to HDAC6 inhibitor Tubacin. **(C)** Immunoprecipitated HA-tagged AKR1C1 from 293FT cells was treated with HDAC inhibitors SAHA (4 μM) and NAM (5 mM) simultaneously for additional 12 h.

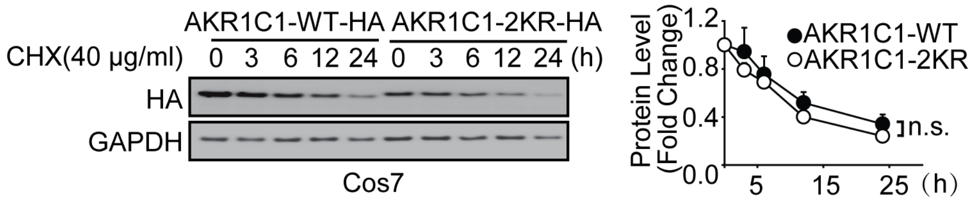

Figure S2. Acetylation imposes little effects on AKR1C1 protein stability. AKR1C1 WT and 2KR mutant exhibited equivalent protein half-life (ANOVA analysis was used).

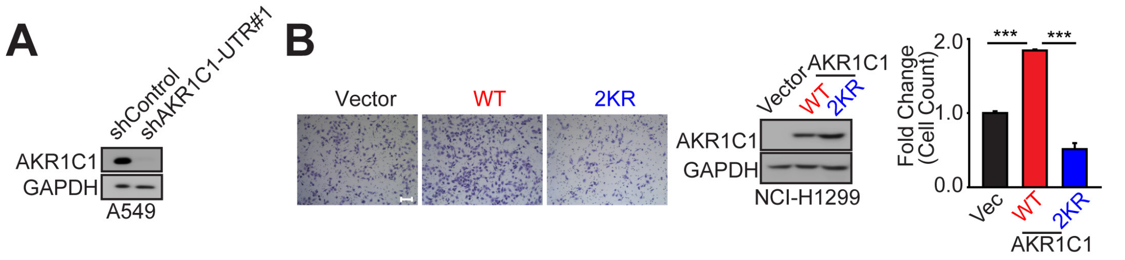

Figure S3. Acetylation of AKR1C1 is crucial for its pro-metastatic ability of NSCLC cells. (A) Western Blotting analysis showing the knockdown efficiency of AKR1C1 targeting the untranslated region 1 (UTR#1). **(B)** Different metastasis phenotypes of NCI-H1299 cells which

were stably expressing Vector, AKR1C1-WT and AKR1C1-2KR. Western Blotting analysis showing the stable transfected efficiency of AKR1C1s. Scale Bar, 200 μm; Statistical significance was determined by Student'st-test (n = 3; ***: $p < 0.001$).

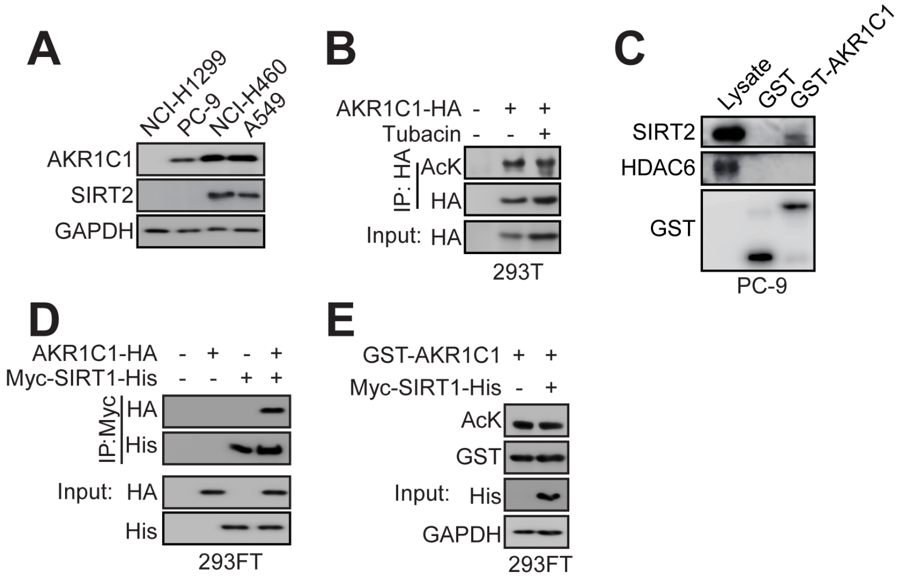

Figure S4. SIRT1 could not deacetylate AKR1C1. (A) The AKR1C1 and SIRT2 protein levels were detected in different NSCLC cell lines. **(B)** HA-tagged AKR1C1 was transfected into 293FT cells for 48 h. Cells were treated with vehicle or Tubacin, and then subjected to analyses of acetylation levels. **(C)** Recombinant GST-tagged AKR1C1 protein was purified and incubated with whole cell lysate of PC-9 cells, and subjected to GST-pull down. **(D)** HA-tagged AKR1C1 and His-tagged SIRT1 were transfected into 293FT cells for 48 h. Immunoprecipitation-western blot for analyzing the interaction between AKR1C1 and SIRT1. **(E)** *In vitro* deacetylation assay was used to evaluate the effect of SIRT1 on AKR1C1, mix recombinant GST-AKR1C1 with purified protein His-tagged SIRT1 in 37°C for 2 h. Western Blot analysis shows the acetylation of GST-AKR1C1 in reaction mixtures.

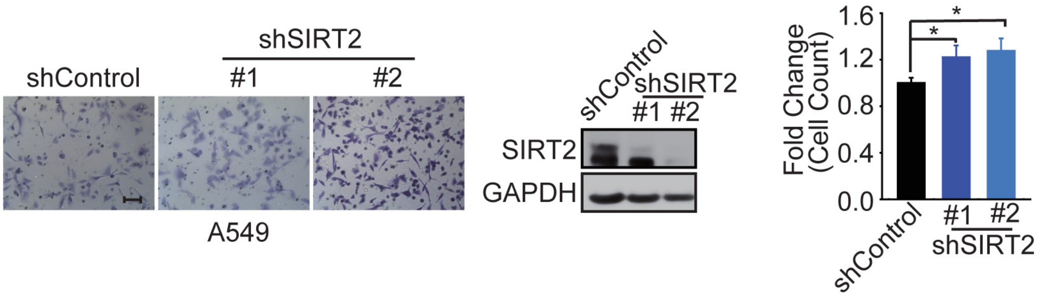

Figure S5. Depletion of endogenous SIRT2 increases the mobility of NSCLC cells. SIRT2 in A549 cells was depleted using shRNA (2 different sequences were introduced), and a transwell assay was utilized to examine the metastatic potential. Scale Bar, 100 μm. Data were analyzedusing two-tailed Student's t-tests. (n = 3; *: $p < 0.05$)

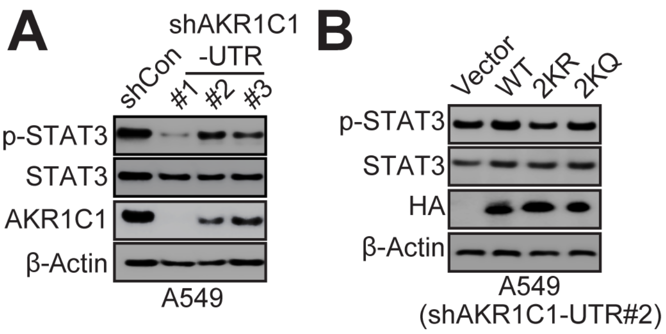

Figure S6. Acetylation deficiency impairs the effect of AKR1C1 to transactivated STAT3 in A549 cells with shAKR1C1-UTR#2. (A) Western Blotting analysis showing the knockdown efficiency of AKR1C1 targeting the untranslated region (UTR) regions. **(B)** Western Blotting analysis of p-STAT3 and STAT3 protein levels in the wild-type and mutant-type (2KR and 2KQ) AKR1C1 transfected in A549 cells (shAKR1C1-UTR#2).

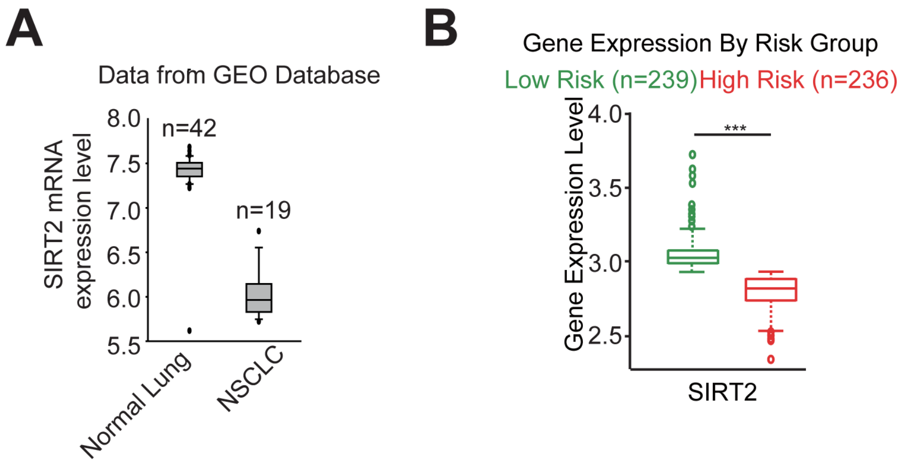

Figure S7. The inverse correlation of SIRT2 expression levels with NSCLC. (A) *SIRT2* mRNA levels in normal lung tissues and tumor samples from NSCLC patients (GSE40275). **(B)** Gene expression levels of *SIRT2* in the low risk (n = 239) and high risk (n = 236) groups of adenocarcinoma of lung cancer patients (TCGA database) (***: $p < 0.001$).

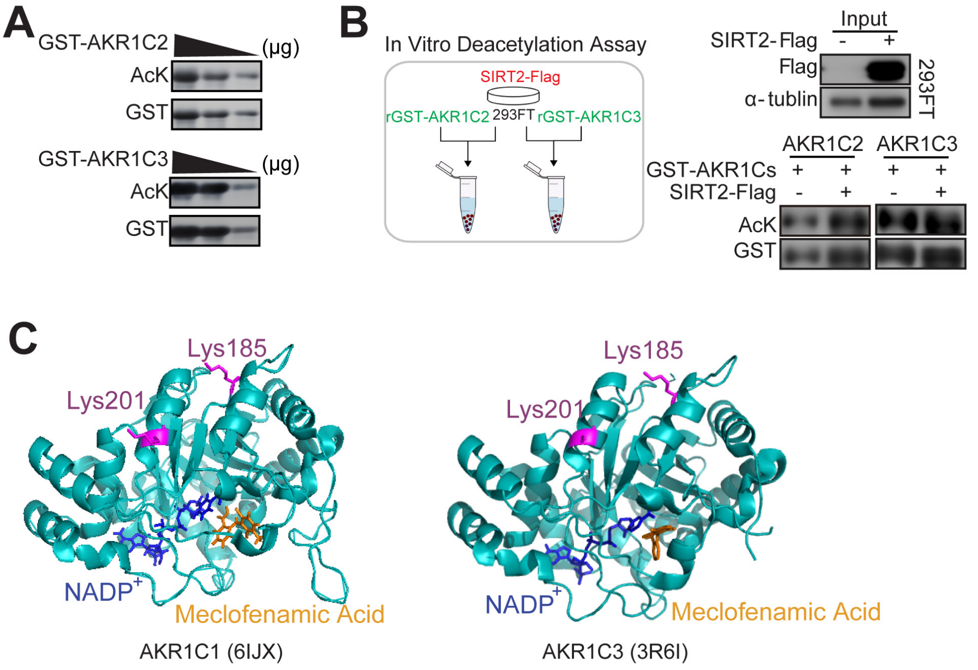

Figure S8. The acetylation of AKR1C2 and AKR1C3 was not regulated by SIRT2. (A) Purified GST-AKR1C2 and GST-AKR1C3 are acetylated. **(B)** *In vitro* deacetylation assay was utilized to determine the influence of SIRT2 on GST-AKR1C2/C3 acetylation. **(C)** The position of lysine 185/201 in the AKR1C1/AKR1C3-NADP$^+$-Meclofenamic Acid complexes (RCSB PDB:

AKR1C1/6IJX, AKR1C3/3R6I). The coenzyme $NADP^+$ and inhibitor Meclofenamic Acid that interact with AKR1C1/C3 are depicted using stick models.

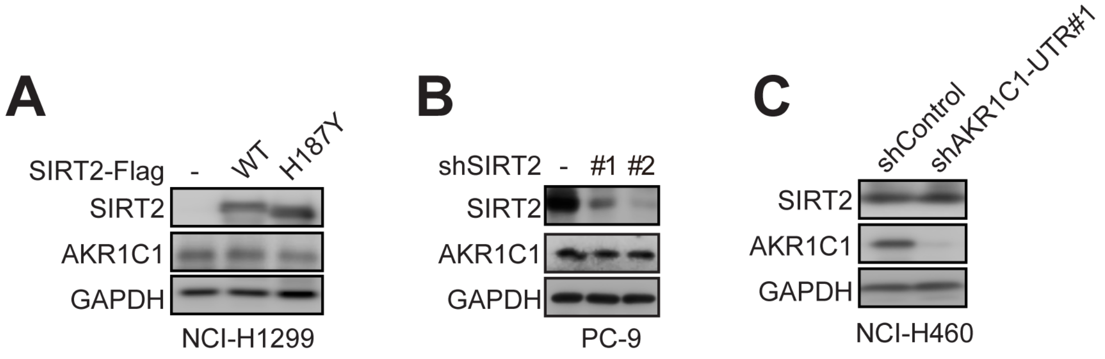

Figure S9. The protein levels of AKR1C1 and SIRT2 are not influenced by each other. (A) Exogenous SIRT2 imposed minimal effect on AKR1C1 protein levels. **(B&C)** Knock-down of SIRT2 (B) or AKR1C1 (C) failed to influence the protein levels of AKR1C1 (B) or SIRT2 (C).

Table S1. Information of plasmid constructs

Plasmid Constructs	Tag	Plasmid Constructs	Tag
pCMV6-AKR1C1-WT	HA	pEGFP-SIRT2-WT	Flag
pCMV6-AKR1C1-2KR	HA	pEGFP-SIRT2-H187Y	Flag
pCMV6-AKR1C1-2KQ	HA	pEGFP-SIRT1-WT	His/Myc
pCMV6-AKR1C1-WT	No-tag	pGEX-4T1	GST
pCMV6-AKR1C1-2KR	No-tag	pGEX-4T1-AKR1C1-WT	GST
pGEX-4T1-AKR1C1-2KR	GST		
